# A rare case of a giant pulmonary solitary fibrous tumor: Diagnostic and therapeutic challenges in the absence of malignancy

**DOI:** 10.1016/j.rmcr.2025.102191

**Published:** 2025-03-14

**Authors:** Nouman Aziz, Waseem Nabi, Sukhrob Makhkamov, Yasmine Elsherif, Adnan Bhat, Sonu Sahni

**Affiliations:** a Wyckoff Heights Medical Center, Brooklyn, NY, USA; b Rocky Vista University College of Osteopathic Medicine, Parker, CO, USA; c Zayed Military Hospital, Abu Dhabi, United Arab Emirates; d University of Florida, Gainesville, USA

## Abstract

**Background:**

Solitary fibrous tumors (SFTs) are rare mesenchymal neoplasms predominantly arising from the pleura but occasionally occurring in extrapleural locations. Pulmonary parenchymal SFTs of extreme size are exceedingly uncommon and present diagnostic and therapeutic challenges.

**Case presentation:**

We report the case of a 60-year-old female with a 14 x 12 × 23 cm pulmonary SFT presenting with a two-week history of productive cough and significant weight loss. Imaging revealed a massive heterogeneous mass in the left lower lobe, compressing lung parenchyma, causing pleural effusion and atelectasis, and raising concerns for metastatic spread to the liver and spleen. Histological examination confirmed the diagnosis of SFT, with spindle-cell morphology and positive immunohistochemical staining for CD34 and STAT6. Despite the tumor's size and compressive effects, histopathology showed no necrosis, pleomorphism, or high mitotic activity, indicative of a non-aggressive phenotype. The patient underwent successful surgical resection via thoracotomy and is under long-term follow-up.

**Discussion:**

This case highlights the unique diagnostic complexity of SFTs, particularly with tumors of exceptional size. While larger SFTs often suggest malignant potential, the absence of typical markers of malignancy in this case emphasizes the unpredictable behavior of these tumors. Imaging and immunohistochemical evaluation are critical for diagnosis, and surgical resection remains the cornerstone of management.

**Conclusion:**

This rare case underscores the importance of thorough diagnostic evaluation and surgical management in addressing giant pulmonary SFTs. Despite their potential for malignant transformation, careful histopathological analysis and multidisciplinary collaboration can guide optimal treatment and long-term surveillance for these unpredictable tumors.

## 1 Introduction

Solitary fibrous tumors (SFTs) are rare mesenchymal neoplasms. They predominantly originate from the pleura but are capable of developing in various extra pleural locations including the lungs, abdomen, and retroperitoneum [1,4]. These account for less than 5 % of pleural tumors and are considered relatively indolent, though approximately 10–20 % exhibit malignant potential [[1], [2], [3]]. SFTs in the parenchyma of the lung, especially those as large as the one presented in this case, are exceedingly rare [1,2]. The diagnosis of SFTs is challenging due to their nonspecific clinical presentation. Symptoms such as cough, chest pain, dyspnea, or weight loss may occur, but many SFTs are discovered incidentally on imaging studies [4].

Tumor size, histological features, and presence of mitotic activity are the primary indicators of its malignant transformation, with tumors larger than 8 cm being particularly concerning [2,3]. Diagnosis of SFTs typically requires histopathological examination and immunohistochemical analysis. Histologically, SFTs are typically composed of spindle cells arranged in a "patternless pattern," often accompanied by thick bands of collagen. Immunohistochemical markers such as CD34 and STAT6 are critical for distinguishing SFTs from other soft tissue tumors [3].

This case report presents a unique case of a patient's tumor measuring an extraordinary 14 x 12 × 23 cm, which was not only associated with an increased risk of malignancy but also caused significant compressive symptoms such as pleural effusion and lung atelectasis. The possibility of metastasis to the liver and spleen added further complexity to this case, illustrating the aggressive potential of such tumors, even in the absence of common histological markers of malignancy, such as necrosis or pleomorphism [3]. Despite the extraordinary size of this patient's tumor and the associated compressive symptoms, pathology revealed a non-aggressive form of the tumor. The histological analysis showed no evidence of necrosis, pleomorphism, or high mitotic activity — markers typically associated with malignancy. This case is significant not only because of the tumor's exceptional size and its potential for aggressive behavior but also due to its clinical presentation with compressive symptoms, making it a unique contribution to the limited body of literature on pulmonary SFTs.

## 2 Case Presentation

A 60-year-old female, previously healthy with no significant medical history, presented with a two-week history of a productive cough and a substantial 30-pound weight loss over the past year. Despite having no personal history of smoking, she had 35 years of secondhand smoke exposure from her partner, raising concerns for a possible malignancy.

On examination, the patient was tachycardic, with a heart rate ranging from 159 to 162 beats per minute. Respiratory examination revealed dullness to percussion in the left lung, with diminished breath sounds at the left apex on auscultation. Mild clubbing of the fingers was also noted. Additionally, a multinodular goiter was visible, with no signs of airway compromise. The chest X-ray showed a large white-out in the left mid to lower lung, suggestive of a pleural effusion, with possible underlying atelectasis or infiltrate. Given the size of the white-out and the patient's significant weight loss, a contrast-enhanced CT scan was obtained.

**Fig. 1:**
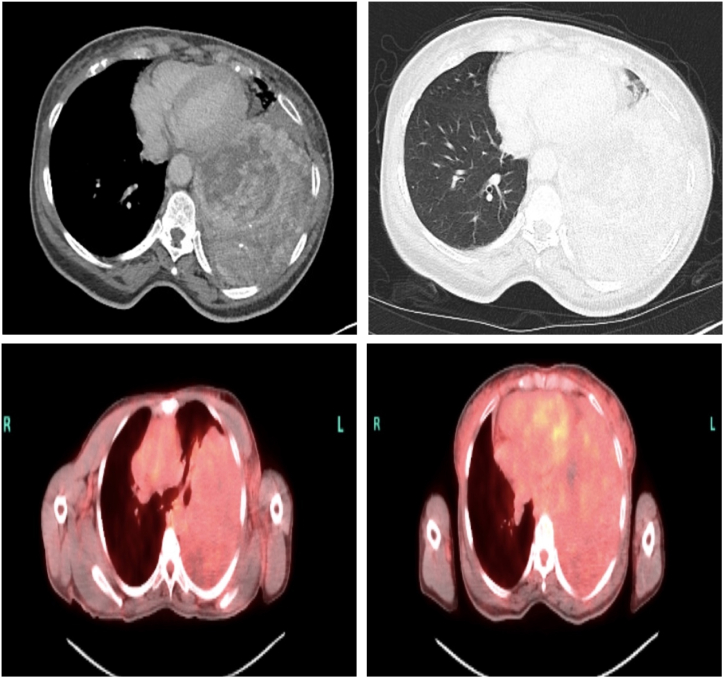
*CT of the chest with and without contrast and PET CT of the chest.* A large mass within the left hemithorax, extending from the mid-lung to the lung base and into the costophrenic angle. The mass is diffusely heterogeneous, with linear enhancing solid components and areas of thick-walled, septated cystic-necrotic changes, particularly in the superior posterior aspect. No significant osseous erosion or invasion of the chest wall is identified.

CT scan of the chest revealed a massive, heterogeneous mass measuring 14 x 12 × 23 cm in the left lower lobe, compressing the lung parenchyma and causing atelectasis of the lingula (Fig. 1). The pulmonary artery was larger than the aorta, suggesting pulmonary hypertension due to mass effect. Additional findings included a multinodular goiter that had narrowed the trachea. The CT also noted multiple lucencies in the liver and spleen, concerning for metastatic spread, although the initial differential also included benign entities like hemangiomas. The size of the tumor, coupled with the imaging features of compressive atelectasis and potential metastasis, made this case especially unique for a solitary fibrous tumor.

**Fig. 2:**
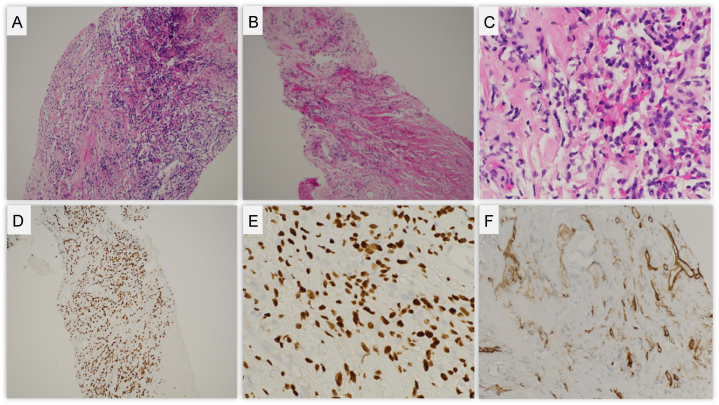
Histopathological and Immunohistochemical Analysis of a Solitary Fibrous Tumor. Top row: Panels A–C show Hematoxylin and Eosin (H&E) stained sections of the tumor at 40x, 100x, and 400× magnification, respectively. Bottom row: Panels D and E demonstrate positive nuclear staining for STAT6 using immunohistochemistry at 100x and 400× magnification respectively. Panel F shows positive CD34 immunohistochemical staining.

A CT-guided biopsy was performed on hospital day 5, obtaining 10 core samples from different regions of the mass. Histological examination of the biopsy confirmed a diagnosis of a solitary fibrous tumor (SFT). The tumor exhibited typical spindle-cell morphology arranged in a patternless architecture, with low mitotic activity. Immunohistochemical staining was positive for CD34 and STAT6, confirming the diagnosis of SFT (Fig. 2). Importantly, there was no evidence of necrosis, atypia, or significant cellular pleomorphism, suggesting that this was a more indolent form of SFT. However, the tumor's large size and compressive effects raised concerns about local invasion and future malignant behavior.

**Fig. 3:**
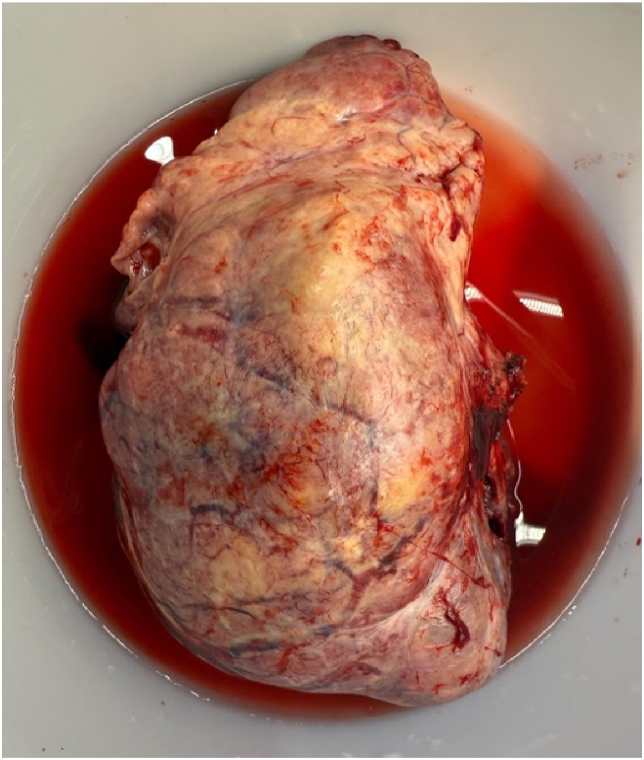
Resected mass.

The patient successfully underwent surgical resection of the lung mass via thoracotomy and is now being followed in a lung cancer clinic for ongoing care (Fig. 3).

## 3 Discussion

This case report details a rare parenchymal pulmonary SFT, notable not only for its unusual location and size but also for the lack of histological evidence typically associated with malignancy, such as necrosis or pleomorphism. Although larger tumors are typically associated with malignant potential, as demonstrated by Magdeleinat et al., the absence of necrosis, atypia, and low mitotic activity in our case suggests a more favorable, benign outcome [4,5]. What sets our case apart is the extreme size of the tumor, coupled with the absence of malignancy, which is atypical for SFTs of this size. This case underscores the unpredictable nature of SFTs and highlights the importance of a multidisciplinary approach to optimize management.

Solitary fibrous tumors are often detected incidentally on chest imaging, as they frequently present without symptoms. As these tumors grow, symptoms such as cough, chest pain, and dyspnea may develop. SFTs also carry a risk of local recurrence, with recurrence rates reported as high as 29 % within five years [15]. Moreover, SFTs have the potential to metastasize, with a 5-year metastasis rate of approximately 34 % [4,15,16]. Tumors larger than 10 cm and those with a high mitotic rate are significant predictors of metastatic spread. In 10 %–20 % of cases, digital clubbing and hypertrophic pulmonary osteoarthropathy have been reported, likely due to excessive release of hyaluronic acid by the tumor [[9], [10], [11]]. Additionally, refractory hypoglycemia, known as Doege-Potter syndrome, occurs in 1 %–2 % of cases, often due to the secretion of insulin-like growth factor II, which is more common in larger or malignant tumors [[12], [13], [14]].

Several case reports have described large SFTs of the lung or pleura, but few have reached the size seen in this case. A similar case involved a 51-year-old male with a 10 cm intrapulmonary SFT. Although the tumor exhibited malignant features such as necrosis, it followed an indolent course after complete surgical resection, with no recurrence or metastasis noted over two years of follow-up [6]. Another case involved a 76-year-old female with a 9.7 cm pleural-based tumor causing compressive symptoms, including hemoptysis and hoarseness due to intratumor bleeding. Despite aggressive clinical behavior, histopathology revealed no malignancy, similar to our case. The patient had a successful recovery post-resection, with no recurrence reported during follow-up [7].

SFTs present diagnostic challenges due to their variable presentation and non-specific symptoms, such as chest pain, cough, or dyspnea, which can be easily mistaken for other thoracic pathologies like bronchogenic carcinoma. Larger tumors, particularly those exceeding 10 cm, add another layer of complexity to management. Imaging modalities, such as CT or MRI, help localize the tumor and assess its size and relationship to surrounding structures, but definitive diagnosis requires histopathological and immunohistochemical evaluation. SFTs are known for their "patternless" histological architecture. In our patient's case, the tumor's spindle-cell morphology and positive staining for CD34 and STAT6 confirmed the diagnosis [2,3]. The benign nature of the tumor in this case, despite its size, is a reminder that large SFTs do not always follow the typical malignant course.

Surgical resection remains the gold standard for managing SFTs, with complete excision being critical to minimizing the risk of recurrence [5,8]. Magdeleinat et al. found that the complete resection was the most significant prognostic factor in malignant SFTs, with incomplete resections leading to recurrence in some cases [5]. For larger tumors or those invading surrounding structures, lobectomy, pneumonectomy, or even chest wall resection may be necessary to achieve clear margins. For our patient, surgical intervention was planned, and given the tumor's size and potential for recurrence, careful monitoring through follow-up imaging was recommended.

Radiotherapy and chemotherapy are generally not indicated for benign SFTs, but in cases where malignancy is suspected or confirmed, adjuvant therapies may be considered. However, the efficacy of these treatments is still debated, with some studies showing limited success [6,8].

This case highlights the unpredictable nature of solitary fibrous tumors, particularly those of significant size. Despite the initial concern for malignancy due to the tumor's large size and compressive symptoms, the final pathology revealed a benign tumor, illustrating the complexity of diagnosing and managing SFTs. Our patient's case underscores the importance of a multidisciplinary approach, careful long-term follow-up, and a tailored treatment strategy based on both clinical presentation and histopathological findings. Future studies are needed to further clarify the factors that predict malignant transformation and to refine treatment and surveillance guidelines for these rare tumors.

## CRediT authorship contribution statement

**Nouman Aziz:** Writing – review & editing, Writing – original draft, Visualization, Validation, Supervision, Software, Resources, Project administration, Methodology, Investigation, Funding acquisition, Formal analysis, Data curation, Conceptualization. **Waseem Nabi:** Writing – review & editing, Writing – original draft, Visualization, Validation, Supervision, Software, Resources, Project administration, Methodology, Investigation, Funding acquisition, Formal analysis, Data curation, Conceptualization. **Sukhrob Makhkamov:** Writing – review & editing, Writing – original draft, Funding acquisition, Formal analysis, Data curation, Conceptualization. **Yasmine Elsherif:** Writing – review & editing, Writing – original draft, Funding acquisition. **Adnan Bhat:** Writing – review & editing, Writing – original draft, Visualization, Validation, Supervision. **Sonu Sahni:** Writing – review & editing, Writing – original draft, Visualization, Resources, Project administration, Funding acquisition.

## Declaration of competing interest

The authors declare that they have no known competing financial interests or personal relationships that could have appeared to influence the work reported in this paper. This case report did not receive any grants or funding from external agencies.

## References

[1] Chang Y.L., Lee Y.C., Wu C.T. (1999). Thoracic solitary fibrous tumor: clinical and pathological diversity. Lung Cancer.

[2] Demicco E.G., Park M.S., Araujo D.M., Fox P.S., Bassett R.L., Pollock R.E., Wang W.L. (2012). Solitary fibrous tumor: a clinicopathological study of 110 cases and proposed risk assessment model. Mod. Pathol..

[3] F.O. Abodunrin, R.B. Killeen, Solitary fibrous tumors, StatPearls (Updated 2024 May 1). In: StatPearls [Internet]. Available from: https://www.ncbi.nlm.nih.gov/books/NBK585038/. 36251813

[4] Gholami S., Cassidy M.R., Kirane A., Kuk D., Zanchelli B., Antonescu C.R., Brennan M. (2017). Size and location are the most important risk factors for malignant behavior in resected solitary fibrous tumors. Ann. Surg Oncol..

[5] Magdeleinat P., Alifano M., Petino A., Le Rochais J.P., Dulmet E., Galateau F., Regnard J.F. (2002). Solitary fibrous tumors of the pleura: clinical characteristics, surgical treatment and outcome. Eur. J. Cardio. Thorac. Surg..

[6] Marcinkevičiūtė K., Jagelavičius Ž., Žurauskas E., Janilionis R. (2024). Giant intrapulmonary solitary fibrous tumor with signs of malignancy. J. Surg. Case Rep..

[7] Tan J.H., Hsu A.A. (2016). Challenges in diagnosis and management of giant solitary fibrous tumour of pleura: a case report. BMC Pulm. Med..

[8] Lin X., Xiang Y., Shi H., Zhang F. (2018). Primary intrapulmonary solitary fibrous tumours. Oncol. Lett..

[9] Mejías-Lafontaine E., Galarza S., Gonzalez-Cancel I. (2021). Digital clubbing as first sign of giant solitary fibrous tumor. A case report. J. Surg. Case Rep..

[10] Auroux M., Adelaide L. (2020, August).

[11] Fridlington J., Weaver J., Kelly B., Kelly E. (2007). Secondary hypertrophic osteoarthropathy associated with solitary fibrous tumor of the lung. J. Am. Acad. Dermatol..

[12] Estrada-Maya J., Montejo J.S., Báez López K.D., Garzón J.C. (2024). Doege-Potter syndrome due to a solitary fibrous tumor of the pleura: a case report. J. Med. Case Rep..

[13] Gohir Q., Ghosh S., Bosher O., Crawford E., Srinivasan K., Moudgil H. (2023). Pleural-based giant solitary fibrous tumour with associated hypoglycaemia: unusual presentation with pulmonary hypertension in a patient with Doege–Potter syndrome. Clin. Med..

[14] Tominaga N., Kawarasaki C., Kanemoto K., Yokochi A., Sugino K., Hatanaka K., Uda S. (2012). Recurrent solitary fibrous tumor of the pleura with malignant transformation and non-islet cell tumor-induced hypoglycemia due to paraneoplastic overexpression and secretion of high-molecular-weight insulin-like growth factor II. Intern. Med..

[15] van Houdt W.J., Westerveld C.M., Vrijenhoek J.E., van Gorp J., van Coevorden F., Verhoef C., van Dalen T. (2013). Prognosis of solitary fibrous tumors: a multicenter study. Ann. Surg Oncol..

[16] O'Neill A.C., Tirumani S.H., Do W.S., Keraliya A.R., Hornick J.L., Shinagare A.B., Ramaiya N.H. (2017). Metastatic patterns of solitary fibrous tumors: a single-institution experience. Am. J. Roentgenol..

